# Symptoms of sleep problems and adherence to dietary guidelines in older women: evidence from the Australian Longitudinal Study on Women’s Health

**DOI:** 10.1017/S1368980023001052

**Published:** 2023-08

**Authors:** Saman Khalesi, Corneel Vandelanotte, Christopher Irwin, Grace E Vincent, Charlotte Gupta, Gita D Mishra

**Affiliations:** 1 Appelton Institute and School of Health, Medical and Applied Sciences, Central Queensland University, Rockhampton, Brisbane & Adelaide, Australia; 2 Menzies Health Institute Queensland and School of Health Sciences and Social Work, Griffith University, Gold Coast, Australia; 3 Australian Women and Girls’ Health Research Centre, School of Public Health, The University of Queensland, Brisbane, Australia

**Keywords:** Sleep, Dietary Guidelines, Women, Lifestyle

## Abstract

**Objective:**

This study examined adherence to dietary guidelines and symptoms of sleep problems (e.g. taking a long time to fall sleep or waking up early) and their associations in a sample of older Australian women (68–73 years of age).

**Design:**

This was a population-based cross-sectional study. Adherence to the dietary guidelines was measured using a validated FFQ and reported as a diet quality score. Symptoms of sleep problems were measured using five questions and a total score was derived. Multivariate linear regression was used to investigate the association between these outcomes, adjusted for the potential confounding influence of demographic (i.e. age and marital status) and lifestyle (i.e. physical activity, stress, alcohol intake, sleep medication use) variables.

**Setting:**

Respondents from the 1946–1951 cohort of the Australian Longitudinal Study on Women’s Health who completed Survey 9 were included.

**Participants:**

Data from *n* 7956 older women (mean age ± sd: 70·8 ± 1·5) were included.

**Results:**

70·2 % reported having at least one symptom and 20·5 % had between 3 and 5 symptoms of sleep problems (mean score ± sd: 1·4 ± 1·4, range 0–5). Adherence to dietary guidelines was poor with an average diet quality score of 56·9 ± 10·7 (range 0–100). Better adherence to dietary guidelines was associated with fewer sleep problem symptoms (*β*: –0·065, 95 % CI: –0·012, –0·005) and remained significant after adjusting for confounding influences.

**Conclusions:**

These findings support the evidence that adherence to dietary guidelines is associated with symptoms of sleep problems in older women.

Poor sleep (disturbed sleep and/or not getting the recommended 7 to 9 h sleep^(1)^) is a major public health problem and a regular occurrence for almost half (45 %) of all Australian adults^(2)^. Poor sleep has important implications, including an increased risk of developing chronic conditions such as hypertension, obesity, diabetes and CVD^(3)^. As a consequence, it has a significant economic and personal impact (i.e. increased health care costs, lost productivity, impaired decision making, workplace absenteeism, road traffic accidents); at an estimated cost of ∼$66 billion annually in Australia^(4)^. In older adults, poor sleep has also been linked with adverse mental health outcomes including depression, dementia and cognitive decline, particularly in women^(5)^.

Many environmental and behavioural factors contribute to the quality of sleep, including lifestyle behaviours such as poor dietary habits, stress and physical inactivity^(6–8)^. From a dietary perspective, consumption of foods high in saturated fat, alcohol and caffeine may lead to poor quality sleep^(6)^. Physical inactivity and stress may also disrupt neuropsychological processes and decrease sleep quality^(9)^. Despite the availability of population-based diet^(10)^ and physical activity^(11)^ guidelines, most Australians do not meet these recommendations^(12)^. In older adults, reduced appetite^(13)^ or compromised ability to prepare foods^(14)^ may affect both the types and amount of food consumed^(13)^, leading to poorer adherence to dietary guidelines. Age-related increases in sleep disorders (e.g. insomnia, sleep-disordered breathing, periodic limb movements in sleep, restless legs syndrome and REM sleep behaviour disorder)^(15)^ psychological stress^(16)^ and decreased physical activity^(17)^ may also influence sleep quality^(9)^. Therefore, older adults often report a high prevalence of symptoms of sleep problems (i.e. short sleep duration, longer time taken to fall asleep, increased number of awakenings, etc.)^(18)^. Older women are also more susceptible to the impact of ageing on sleep compared with older men^(5)^. Hormonal changes post-menopause can lead to circadian disruption and sleep architectural changes^(19)^. These may explain the gender differences in the relationship between diet and sleep in the older populations previously reported^(20,21)^. Given that there is an increasing proportion of older Australians in the population^(22)^, and that older women are more susceptible to the consequences associated with poor sleep, it is important to understand the relationship between dietary behaviours and sleep in this specific demographic; including the influence of contextual factors (i.e. physical activity, stress, alcohol intake) on this relationship.

Therefore, the aim of this study was to explore the association between adherence to dietary guidelines and symptoms of sleep problems (controlling for contextual factors), in a sample of older Australian women (68–73 years of age). Findings from this study will inform the development of interventions and recommendations aimed at improving sleep quality and dietary intake, as well as the overall wellbeing of older women in Australia.

## Methods

### Study participants and design

This study involved an analysis of data collected in the Australian Longitudinal Study on Women’s Health (ALSWH). This population-based longitudinal study was established in 1996 to monitor the health of Australian women in three age groups according to birth year (1973–1978 cohort, 1946–1951 cohort, 1921–1926 cohort) with a fourth age group (1989–1995 cohort) added in 2013. Details of the study and methods are available at www.alswh.org and have been published elsewhere^(23)^. Briefly, women were randomly selected from the Medicare database to ensure a nationally representative sample was obtained. Ethical approval was obtained from the Human Research Ethics Committees of the University of Newcastle (approval number: h–076–0795) and the University of Queensland (approval number: 200400224). Participants were invited to complete a survey containing questions on physical and mental health, well-being, lifestyle behaviours, life satisfaction, medical history and demographic characteristics.

The current study used data from the 1946 to 1951 cohort (survey 9, completed in 2019). The response rate from the first survey was 58 %. Information collected on dietary intake, symptoms of sleep problems, stress, physical activity, alcohol intake, sleep medication use and demographic characteristics were included for analysis.

### Adherence to dietary guidelines

Dietary intake was assessed using the Commonwealth Scientific and Industrial Research Organisation (CSIRO) Healthy Diet Score tool^(24)^. This is a validated short FFQ containing thirty-eight questions about the quantity, quality and variety of foods consumed. For food quantity, consumption frequency was converted to the daily number of serves for the following food groups: ‘fruit’, ‘vegetables and legumes/beans’, ‘grains (cereals)’, ‘meat and alternatives’, ‘dairy and alternatives’ and ‘discretionary foods’. For food quality, consumption frequency of wholegrains, reduced fat dairy and water (fluid), meat trimming and the types of fat used were assessed. This allowed the calculation of an overall diet score (out of 100)^(24)^, based on compliance with the Australian Dietary Guidelines (ADG)^(25)^, with higher scores representing better adherence and diet quality^(24)^.

### Symptoms of sleep problems assessment

Symptoms of sleep problems were explored using five questions asking about the presence of any of these typical sleeping problems: ‘waking up in the early hours of the morning’; ‘lying awake for most of the night’; ‘taking a long time to get to sleep’; ‘worry keeping you awake at night’ and ‘sleeping badly at night’. These questions target symptoms such as difficulty falling asleep and restless sleep; key indicators of poor sleep quality in older populations^(26)^. A score was allocated to each of the five questions (1 = ‘Yes’, 0 = ‘No’ responses) and an overall score was determined from the sum of individual scores (max 5), with acceptable internal consistency reliability (Cronbach’s *α* = 0·643, *n* 5). Higher scores suggest more symptoms of sleep problems^(26)^.

### Assessment of demographic and contextual factors

Stress, physical activity, alcohol intake, sleep medication use and demographic characteristics of age and marital status were also explored. Perceived stress was assessed using a 10-item Perceived Stress Questionnaire scale previously developed and validated in the ALSWH^(27)^. It includes questions relevant to different aspects of life (i.e. own health, living arrangement, money, health of family members, work/employment, study, relationship with parents, relationship with partner/spouse, relationship with children, relationship with other family members)^(27)^. Participants rated each aspect according to the Likert categories ‘Not applicable’, ‘Not at all stressed’, ‘Somewhat stressed’, ‘Moderately stressed’, ‘Very stressed’ and ‘Extremely stressed’, which were scored as 0, 0, 1, 2, 3 and 4, respectively. An overall stress score was calculated using the sum of perceived stress scores across all aspects^(27)^.

Physical activity status was assessed using a modified version of Active Australia Survey^(28)^, which has previously demonstrated acceptable reliability and validity^(29)^. Participants’ frequency and duration of walking, moderate and vigorous physical activities per week were scored based on MET-minutes/week and categorised as ‘Nil/Sedentary’ (representing 0 to 10 min of moderate activity per week), ‘Low ’ (representing 11 to 150 min of moderate activity per week), ‘Moderate’ (representing 151 to 300 min of moderate activity per week) and ‘High’ (representing > 300 min of moderate activity per week)^(30)^. Moderate activity of at least 30 min 5 times per week (> 150 min per week, representing ‘Moderate’ and ‘High’ category of physical activity status) is considered as the threshold of meeting physical activity guidelines^(11,30)^.

Alcohol intake was measured using a questionnaire derived from the Australian National Health and Medical Research Council (NHMRC) guidelines^(31)^ on the frequency and quantity of alcohol intake and was categorised as ‘non-drinker’, ‘Low risk (≤ 2 standard drinks/d)’, ‘Risky (3–4 standard drinks/d)’ and ‘High risk (≥ 5 standard drinks/d)’^(32)^.

Sleep medication use was assessed using one question on using medication to help sleep. Demographic data of age, living arrangement and marital status were also included.

### Statistical analysis

Statistical analysis was performed using SPSS (Version 26, IBM Corp, New York). Descriptive statistics were used to determine the mean ± sd or frequency (%) for diet scores, symptoms of sleep problems, stress score, physical activity status and patterns of alcohol consumption. The association between scores for diet quality and symptoms of sleep problems was investigated using multivariate linear regression, either unadjusted (model 1) or adjusted for the potential confounding influence of demographic (i.e. age and marital status) and lifestyle (i.e. physical activity, stress, alcohol intake, sleep medication use) variables (model 2). Outcomes were presented as regression (standardised) coefficients with 95 % CI. Linear regression assumptions were checked prior to analysis. The linearity of the relationship was investigated via a scatterplot and correlation analysis. A *P*-value of < 0·05 was accepted as statistical significance.

## Results

Data available for the current study included *n* 7956 women aged 68–73 years (mean ± sd: 70·8 ± 1·5). Demographic characteristics of the participant cohort are presented in Table 1. Most participants were living with partners, family members or others (76·5 %). Less than half (42·3 %) of participants reported not being active or having low physical activity, and the majority (95·2 %) reported not drinking alcohol or having a low risk of drinking alcohol (≤ 2 standard drinks/d). Participants’ mean stress score was 0·4 ± 0·4 (representing not or somewhat stressed).

**Table 1:** Demographic characteristics of participants

Characteristics	Mean or Frequency	sd or %
Age (years) (*n* 7956)*	70·8	1·5
Marital status (*n* 7806)		
Married/Living with partner	5374	68·8
Divorced/Separated	1172	15·0
Widowed	1051	13·5
Never Married	209	2·7
Stress (*n* 7846)* (0 = not stressed; 4 = extremely stressed)	0·4	0·4
Physical Activity (*n* 7597)		
Not active	1319	17·4
Low	1892	24·9
Moderate	1672	22·0
High	2714	35·7
Alcohol risk (*n* 7275)		
Non-drinker	623	8·6
Low risk (≤ 2 standard drinks/d)	6303	86·6
Risky (3–4 standard drinks/d)	333	4·6
High risk (≥ 5 standard drinks/d)	16	0·2

* Presented as mean ± sd.

At the time of the survey, the majority of participants (70·2 %) reported having at least one symptom of sleep problems and 20·5 % had between 3 and 5 symptoms. The mean of the symptoms of sleep problem score was 1·4 ± 1·4 (range 0 to 5). The prevalence of type of symptoms varied, but more than half (54 %) of the participants reported ‘waking up in the early hours of the morning’, almost one-third (31·4 %) reported they ‘took a long time to go to sleep’ and 28·1 % reported they ‘sleep badly at night’. In 14·2 % ‘worry kept them awake’, and another 14·1 % reported they ‘lie awake for most of the night’. However, the majority (*n* 6287, 83 %) reported not taking any medications in the past 4 weeks to help with sleep.

The average number of serves consumed from the five core food groups and discretionary foods, including the age- and sex-associated (i.e. 70+ years) serve recommendations according to the ADG^(10)^ are presented in Table 2. Participants had lower intakes (number of serves) for ‘dairy and alternatives’ and ‘vegetables and legumes/beans’, and higher intakes for ‘grains (cereals)’ and ‘discretionary foods’ compared with the ADG recommendations. This resulted in an average diet quality score of 56·86 ± 10·73 (*n* 7141; range from 0 to 100).

**Table 2:** Number of serves of ADG-based food groups and their comparison between dietary patterns

	ADG recommended serves (51–70 and 70+ age range)	Number of serves consumed		Meeting ADG	
		Mean	sd	*n*	%
Dairy and alternatives	4	1·43	1·09	50	0·7
Fruit	2	1·68	1·08	2598	36·4
Vegetables and legumes/beans	5	3·97	2·64	1976	27·7
Meat and alternatives	2	2·00	1·12	2854	40·0
Grain (cereal) foods	3–4	3·74	2·58	1876	26·3
Discretionary foods	0–2 ½	2·73	2·01	72	1·0

ADG: Australian Dietary Guidelines – serves based on the recommended average daily number of serves for women aged 51–70 and 70+ years^(10)^.

**Fig. 1:**
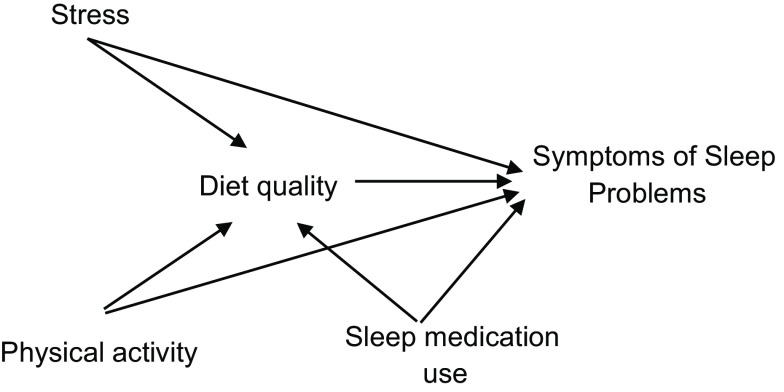
Associations between scores for diet quality and symptoms of sleep problems confounding by the influence of stress, physical activity and sleep medication use

The association between diet score and symptoms of sleep problems is presented in Fig. 1. A higher diet quality score (better adherence to ADG) was associated with lower symptoms of sleep problem score (*β*: –0·065, 95 % CI: –0·012, –0·005, *P*-value < 0·001). The association remained significant when the model was adjusted for the confounding influence of demographic (i.e. age and marital status) and lifestyle (i.e. stress, alcohol intake, physical activity, sleep medication use) variables (*β*: –0·025, 95 % CI: –0·006, –0·000 *P*-value 0·035). The influence of using sleep medication, not being physically active and higher stress scores (but not risky alcohol intake or demographic variables) were significant in the association between higher diet score and lower symptoms of sleep problems (*P*-values < 0·001). Linear regression assumptions were met with no multicollinearity observed between independent variables and sleep score (correlation *r* < 0·7). Probability–probability (P–P) plot showed normal distribution. Cook’s distance was also < 1. However, the dependent variable (sleep score) was not normally distributed (Kolmogorov–Smirnov = 0·246, *P*-value < 0·01). Given the large sample size (*n* > 3000), linear regression is still a valid test despite the non-normally distributed dependent variable^(33)^.

## Discussion

This study explored the association between adherence to dietary guidelines and symptoms of sleep problems in a sample of older Australian women (68–73 years of age) using data from the ALSWH. The data indicated low adherence to dietary guidelines and a high prevalence of having at least one symptom of sleep problems in this population group. The analyses suggest that better adherence to dietary guidelines is associated with fewer self-reported symptoms of sleep problems, even after controlling for the influence of stress, physical inactivity, alcohol intake and sleep medication use.

Most Australians (irrespective of age and gender) do not meet the recommended intakes outlined in the ADG for the core food groups (i.e. fruit, vegetables, grains, lean meats and alternatives, dairy and alternatives) and over-consume discretionary (‘junk’) foods^(34,35)^. Adherence to dietary guidelines is important for healthy ageing and to maintain quality of life and good mental health^(36)^. However, poor adherence to dietary guidelines has been previously reported in studies of older Australians^(37,38)^. The current study also confirms a similar trend in a relatively large sample of older Australian women. Individual and environmental changes that occur with ageing may explain this poor adherence to dietary guidelines^(13)^. Many older adults experience a decline in appetite due to changes in the digestive system, increased prevalence of illnesses and medication use and psychological changes^(13)^. In older women, hormonal changes post-menopause may exacerbate the psychological and mood changes^(19)^. Reduced mobility and financial constraints may also affect the accessibility and affordability of food in older ages^(39)^. More older women, compared with men are likely to live alone or in cared accommodation^(40)^, further influencing their food choices and dietary quality. While living arrangement was controlled in the analysis of the current study, almost one-fourth (23·5 %) of the older women who participated in this study reported living alone.

The analysis in the present study also indicates that symptoms of sleep problems are highly prevalent in older Australian women. Indeed, the majority (87 %) of the cohort surveyed in the ALSWH reported having at least one sleep problem symptom. This is consistent with data reported in a 2016 survey of Australian adults, with more than half (61·1 %) of adults aged 65 years and older self-reporting to have at least one sleep problem symptom; with a higher prevalence of symptoms in females compared to males in all age groups^(41)^. A possible explanation may relate to age-associated physiological changes in sleep architecture and normal circadian rhythms^(15,19)^. In older women, this is exacerbated by the age-related decrease in melatonin secretion and increase in the onset of cardiovascular, metabolic and mood disorders^(19)^. Other individual and environmental factors such as feeling safe, noise and light, living arrangements, stress and lifestyle/behavioural factors can also influence sleep quality and architecture^(42,43)^.

From a dietary perspective, a bidirectional relationship between dietary choices and sleep quality exists. High intakes of discretionary (‘junk’) foods^(43)^ and lower adherence to healthy dietary patterns (e.g. Mediterranean^(44)^) have been associated with poorer sleep quality^(45)^. Furthermore, diets rich in carbohydrates, dairy foods and certain nutrients and phytonutrients have been linked to improved sleep outcomes^(43,46)^. On the other hand, poor-quality sleep has been linked with increased intake of energy-dense (i.e. discretionary) foods^(47,48)^.

Several mechanisms are proposed for the relationship between diet and sleep. Dietary intake of the amino acid tryptophan (abundant in dairy, beans, chicken, nuts, etc.) has the ability to convert to serotonin and consequently the sleep-promoting hormone, melatonin, which plays a major role in reducing sleep problems and improving sleep quality^(46)^. Carbohydrates (especially low glycemic and complex carbohydrates) in food can also help with the availability of tryptophan to cross the blood-brain barrier and convert to melatonin^(21,49)^. In addition, lower consumption of fibre and higher intakes of saturated fats and sugar (characteristics of discretionary foods) have been associated with increased sleep problems and lighter sleep^(50)^. Conversely, sleep restriction has been linked with an increase in the hunger hormone, ghrelin^(48)^, a decrease in the appetite-suppressing hormone, leptin^(47,51)^ and stimulation of the brain reward centre^(51)^. This may lead to an increase in the number and portion size of meals consumed and desire for energy-dense (i.e. discretionary) foods. As such, adherence to dietary guidelines (that promote healthier food intake behaviours) is likely to be associated with better quality sleep (and vice versa). Results presented in the current study align with this premise, with the regression analyses suggesting that a higher diet quality score is inversely associated with symptoms of sleep problem in older Australian women, even after controlling for other lifestyle and behavioural factors such as stress, alcohol intake, physical inactivity and sleep medication.

Our results also suggest that physical inactivity and higher levels of stress positively influence the association between diet and symptoms of sleep problems in older women. Physical activity can increase energy expenditure, improve neuropsychological performance and reduce mood and stress, ultimately improving sleep quality^(52)^. In a sample of post-menopausal women, a 12-week increase in the amount of walking improved sleep quality and reduced sleep disturbances/problems^(53)^. Physical activity is also associated with improved mental and emotional health and reduced stress and anxiety in older adults^(54)^. Stress can lead to nervous system hyperactivation, overriding the normal sleep-promoting processes^(55)^. It can also lead to depression and anxiety, further reducing sleep quality^(56)^.

Results for the current study are based on a large sample of older Australian women (*n* 7956), which increases the generalisability of the findings. However, the current study also has some limitations. Outcomes are based on self-reported information, which may not accurately represent an individual’s actual measures. The study was also limited to women aged between 68 and 73 years, limiting the generalisability of the findings to all older Australians (i.e. 65 years and older). Also, while the sleep questions were chosen to target common symptoms of sleep problems in older adults and had acceptable internal consistency reliability, they do not constitute a validated scale. Future studies using validated scales, such as Pittsburgh Sleep Quality Index^(57)^ in this population are required to support the findings of this study. Other individual factors, such as sleep hygiene and environmental factors such as light, noise and distractions, can also confound the relationship between diet quality and symptoms of sleep problem, which were not accounted for in the available data. Furthermore, since the findings are based on observational data, a cause–effect relationship cannot be determined.

### Conclusion

Symptoms of sleep problems are prevalent in older Australian women and their adherence to dietary guidelines is poor. There is a link between better adherence to dietary guidelines and lower likelihood of reporting symptoms of sleep problems in this population, even after controlling for the influence of stress, physical inactivity, alcohol intake and sleep medication use. Future public health guidelines and interventions aimed at improving diet and sleep quality in older women should consider the bidirectional relationship between the two and assess and monitor their adherence to the guidelines to improve general health and well-being in this population.
